# Molecular diagnostic testing for Klinefelter syndrome and other male sex chromosome aneuploidies

**DOI:** 10.1186/1687-9856-2012-8

**Published:** 2012-04-23

**Authors:** Karl Hager, Kori Jennings, Seiyu Hosono, Susan Howell, Jeffrey R Gruen, Nicole R Tartaglia, Henry M Rinder

**Affiliations:** 1 JS Genetics, Inc, 2 Church St. South, B-05, New Haven, CT, 06519, USA; 2 Children’s Hospital Colorado, Aurora, CO, 80045, USA; 3 Yale Child Health Research Center, Yale University School of Medicine, New Haven, CT, 06520, USA; 4 Department of Pediatrics, Yale University School of Medicine, New Haven, CT, 06520, USA; 5 Department of Genetics, Yale University School of Medicine, New Haven, CT, 06520, USA; 6 Investigative Medicine Program, Yale University School of Medicine, New Haven, CT, 06520, USA; 7 Department of Pediatrics, University of Florida, Gainesville, FL, 32610, USA; 8 Department of Laboratory Medicine, Yale University, New Haven, CT, 06510, USA

**Keywords:** Pyrosequencing, Sex chromosome aneuploidy, Klinefelter (47,XXY) syndrome, 47,XYY syndrome, 48,XXYY syndrome, 48,XXXY syndrome, Male infertility

## Abstract

**Background:**

Male sex chromosome aneuploidies are underdiagnosed despite concomitant physical and behavioral manifestations.

**Objective:**

To develop a non-invasive, rapid and high-throughput molecular diagnostic assay for detection of male sex chromosome aneuploidies, including 47,XXY (Klinefelter), 47,XYY, 48,XXYY and 48,XXXY syndromes.

**Methods:**

The assay utilizes three XYM and four XA markers to interrogate Y:X and X:autosome ratios, respectively. The seven markers were PCR amplified using genomic DNA isolated from a cohort of 323 males with aneuploid (n = 117) and 46,XY (n = 206) karyotypes. The resulting PCR products were subjected to Pyrosequencing, a quantitative DNA sequencing method.

**Results:**

Receiver operator characteristic (ROC) curves were used to establish thresholds for the discrimination of aneuploid from normal samples. The XYM markers permitted the identification of 47,XXY, 48,XXXY and 47,XYY syndromes with 100% sensitivity and specificity in both purified DNA and buccal swab samples. The 48,XXYY karyotype was delineated by XA marker data from 46,XY; an X allele threshold of 43% also permitted detection of 48,XXYY with 100% sensitivity and specificity. Analysis of X chromosome-specific biallelic SNPs demonstrated that 43 of 45 individuals (96%) with 48,XXYY karyotype had two distinct X chromosomes, while 2 (4%) had a duplicate X, providing evidence that 48,XXYY may result from nondisjunction during early mitotic divisions of a 46,XY embryo.

**Conclusions:**

Quantitative Pyrosequencing, with high-throughput potential, can detect male sex chromosome aneuploidies with 100% sensitivity.

## Introduction

Klinefelter syndrome (KS, also known as 47,XXY) and 47,XYY syndrome are the two most common sex chromosome aneuploidies in humans with prevalence of approximately 1 in 600–1000 males [1-4]. Individuals with KS are usually tall adolescents and adults who have hypergonadotrophic hypogonadism with small testicles. However, the KS phenotype is highly variable and individuals may not show these physical features to a degree that distinguishes them from the general male population. Males with 47,XYY are also taller than average, but in contrast to KS, they usually do not have phenotypic characteristics to differentiate them from 46,XY males. Compared to 46,XY males, individuals with KS or 47,XYY syndrome exhibit a greater incidence of behavioral problems, psychiatric disorders and neuropsychological characteristics including developmental delays and difficulties in cognitive, verbal and social skills [5]. Yet individuals with both syndromes often fail to be ascertained. Newborn screening studies estimate that only 25% of all individuals with KS, and 10% of all individuals with 47,XYY are diagnosed during their lifetime [6,7].

More complex male sex chromosome aneuploidies, such as 48,XXYY and 48,XXXY, are less common than KS with prevalences ranging from 1 in 18,000 to 1 in 100,000 or greater [1]. While some phenotypic characteristics of 48,XXYY and 48,XXXY syndromes overlap with KS, the unique and significant differences in physical appearance, cognitive function, social and adaptive skills observed in affected individuals differentiate these aneuploidies from KS [8,9].

The gold standard for detection of chromosome aneuploidies is karyotype analysis, an invasive, time-consuming and labor-intensive process. Yet despite the widespread availability of karyotyping, most males with sex chromosome aneuploidy are never diagnosed during their lifetime [6,7]. Thus, the development of more convenient methods for detection of sex chromosome aneuploidies should facilitate identification of these individuals, allowing them to receive early evaluation and therapeutic intervention as indicated. To address this need, we developed a two-stage Pyrosequencing based assay which measures Y:X and X:autosome chromosome ratios. Using this approach, we demonstrated 100% sensitivity in the identification of males with sex chromosome aneuploidy.

## Materials & methods

### DNA samples

De-identified karyotype-confirmed DNA samples from individuals with male sex chromosome aneuploidies (n = 117) were obtained from Children's Hospital Colorado and the UC Davis MIND Institute (Dr. Flora Tassone). Additional de-identified karyotype-confirmed DNA samples from subjects with 45,X (n = 1), 46,XX (n = 4), 47,XXX (n = 11), and 46,XY (n = 206) were provided by the above sources, plus the Yale Cytogenetics Lab (Dr. Peining Li) and the Genetics Diagnostic Lab of Children’s Hospital Boston (Dr. Bai-Lin Wu). Before use, the DNA samples were diluted 20-fold with nuclease-free water. The concentration of diluted DNA was determined by real time PCR using the human specific probe WIAF699 as described [10]. Only samples with a concentration of diluted DNA ≥1 ng/μl were used as templates for PCR of the XYM and XA markers (see below).

### DNA isolation from buccal swabs

Buccal swabs were obtained from patients of Children’s Hospital Colorado after informed consent. DNA was isolated from buccal cells using the Qiagen EZ1 robot and EZ1 DNA tissue kit according to the manufacturer’s protocol. Extracted DNA was quantified by real time PCR as above.

### Assay design

Pyrosequencing (PSQ) assays were designed to interrogate two types of markers. The first class of markers, designated XYM, consisted of regions of the X and Y chromosomes with nearly identical sequence that differ by a chromosome-specific biallelic single nucleotide polymorphism, such that one allele is present only on the X chromosome and the other allele is located on the Y chromosome. Candidate sequences were identified by examining closely related genes present on both X and Y chromosomes outside of the terminal pseudoautosomal regions (see Table 1 of reference [11] and Table 2 of reference [12]). BLAST [13] searches of the human reference genome sequence were used to confirm a single match to the X and Y chromosomes for all sequences entered into the PSQ assay design software (version 1.0.6).

**Table 1:** Statistical values calculated from the percent Y allele signals of the three XYM markers for samples grouped together by karyotype

**Karyotype**	**47, XXY (n = 42)**			**47, XYY (n = 26)**			**48, XXXY (n = 4)**			**48, XXYY (n = 45)**			**46, XY (n = 206)**		
**Marker**	**XYM1**	**XYM2**	**XYM3**	**XYM1**	**XYM2**	**XYM3**	**XYM1**	**XYM2**	**XYM3**	**XYM1**	**XYM2**	**XYM3**	**XYM1**	**XYM2**	**XYM3**
**Average**	32.3	33.3	33.7	63.7	62.5	64.6	24.4	26.7	26.0	47.4	48.1	49.3	48.2	49.7	49.3
**Median**	32.3	33.4	33.7	63.6	62.2	64.7	24.1	26.7	25.7	47.6	48.0	49.4	48.2	49.8	49.2
**Std Dev**	1.53	2.56	1.39	1.55	1.53	0.83	1.54	0.57	0.90	1.17	2.70	1.15	1.46	1.61	1.55
**Maximum**	38.9	40.6	39.0	67.8	65.9	66.8	26.4	27.3	27.3	50.6	58.3	51.8	51.9	53.2	53.3
**Minimum**	30.1	27.9	31.0	61.5	57.8	62.3	23.0	25.9	25.3	45.4	44.2	46.1	43.1	41.1	42.8

**Table 2:** Statistical values calculated from the percent X allele signals of the four XA markers for samples grouped together by karyotype

**Karyotype**	**48, XXYY (n = 45)**				**46, XY (n = 206)**			
**Marker**	**XA1**	**XA2**	**XA3**	**XA4**	**XA1**	**XA2**	**XA3**	**XA4**
**Average**	56.8	54.1	48.5	50.4	42.3	35.3	33.0	32.0
**Median**	56.4	54.0	48.9	50.8	42.1	35.1	33.3	31.7
**Std Dev**	1.74	2.57	2.43	3.47	2.51	2.03	2.99	3.56
**Maximum**	59.7	59.1	51.1	57.8	60.2	42.6	37.2	51.3
**Minimum**	52.2	49.5	35.3	43.8	35.5	27.4	0.0	20.8

The second class of markers, designated XA, represent regions of the X chromosome that are nearly identical with an autosome except for a single chromosome-specific base. Candidate sequences for assay design were identified by BLAST searching the human genome reference sequence with the set of all X-chromosome transcripts obtained from the ENSEMBL database [14]. High scoring matches where at least 200 bases were >95% identical with one locus on the X-chromosome and one on an autosome were used to generate consensus sequences for assay design by the PSQ software.

PCR and extension primers for high scoring assays were synthesized using standard methods by the W. M. Keck Foundation Biotechnology Resource Lab of Yale University. One of the PCR primers for each assay was labeled at the 5´ end with biotin. For all assays, the extension primer had the same orientation as the forward PCR primer and is complementary to the biotinylated template strand generated with the reverse PCR primer.

### PCR and pyrosequencing

A minimum of 2.5 ng genomic DNA was used as template in a 25 μl PCR reaction. Each PCR reaction contained 1 X Hotstar buffer (Qiagen), 2.5 mM MgCl_2_, 200 μM each dNTP, 1 μM each PCR primer, 0.5 U Hotstar Taq Plus (Qiagen). Reactions were performed as follows: initial incubation of 5 min at 95°C; followed by 45 cycles of 30 sec at 95°C, 45 sec at 56°C, and 60 sec at 72 °C; then 5 min at 72 °C and a final hold at 4°C. Upon completion of PCR, the biotinylated template strand was purified using Streptavidin-Sepharose and the Filter Prep tool (Qiagen) according to the manufacturer’s instructions. The resulting single stranded template was annealed to the appropriate extension primer and Pyrosequencing was performed using Pyromark Q96 reagents and PSQ96MA instrument (Qiagen). The allele percentage was calculated by the PSQ software (version 2.1) and exported for analysis by Microsoft Excel.

## Results

### Principle of assay

**Figure 1:**
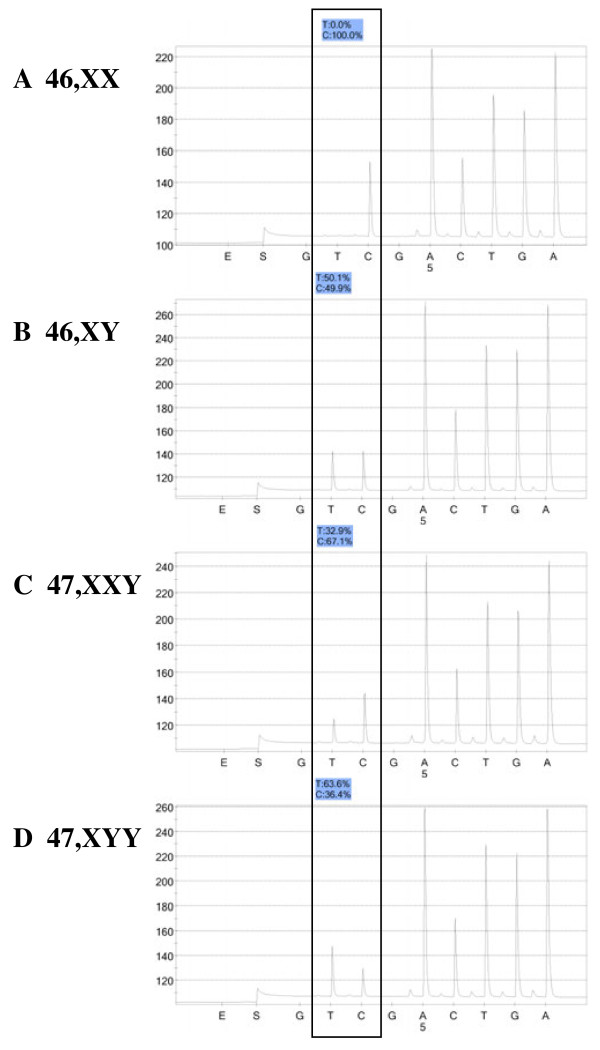
**Pyrograms for DNA from a 46,XX female (A), a 46,XY male (B), a 47,XXY KS male (C), and a 47,XYY male (D) using the XYM3 assay.** The box encloses the two nucleotide dispensations which define the C/T SNP; the C-allele is derived from the X-chromosome and the T-allele is from the Y-chromosome. The percent of each allele is shown in the shaded box above each pyrogram. The y-axis depicts the intensity of light signal in arbitrary units and the x-axis shows the time of addition of Pyrosequnecing enzymes (E), substrates (S), and each individual nucleotide dispensation (A, C, G, or T).

**Figure 2:**
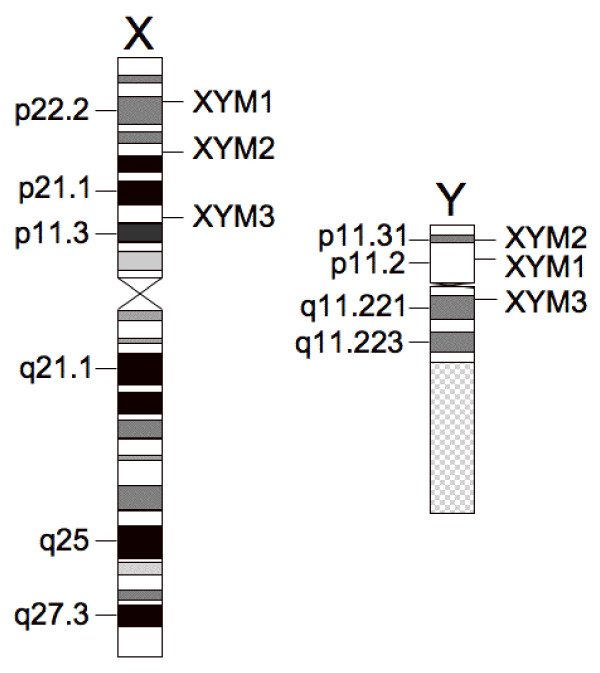
**Locations of the XYM markers on the X and Y chromosomes.** X and Y chromosome ideograms are drawn to scale using data from the UCSC genome browser. X chromosome is 154.9 Mb and Y chromosome is 57.8 Mb.

The assay measures both Y:X and X:A (X:Autosome) chromosome ratios by Pyrosequencing, a quantitative short-read DNA sequencing technology [15]. For the first step, three XYM loci are PCR amplified with specific primer pairs and the resulting amplicons subjected to Pyrosequencing, yielding the percent of Y-chromosome-specific allele signal for each locus and hence, the ratio of the Y and X chromosomes. Figure 1 shows Pyrosequencing data using the XYM3 marker with DNA from a 46,XX female (A), 46,XY male (B), a 47,XXY individual (C), and a 47,XYY subject (D). The percent Y allele signal has close agreement with the value predicted for each karyotype. The locations of the three XYM markers on the X and Y chromosomes are shown in Figure 2.

In the second step of the assay, Pyrosequencing of the four XA markers determines the X:autosome (X:A) ratio, which permits differentiation between individuals with 46,XY and 48,XXYY karyotypes.

### Overall performance of XYM markers

To evaluate the ability of the three XYM markers to detect sex chromosome aneuploidies, PCR and Pyrosequencing were performed on 339 DNA samples from females (n = 16) and males (n = 323), with the technician blinded to each individual’s karyotype. Following completion of Pyrosequencing, the karyotype of each sample was matched to the data for all three markers. All samples from phenotypic females, including individuals with a 46,XX karyotype (n = 4) and those with sex chromosome aneuploidies (45,X, n = 1 and 47,XXX, n = 11), did not display detectable Y allele for any of the three XYM markers (maximum Y allele signal of 1.9%).

**Figure 3:**
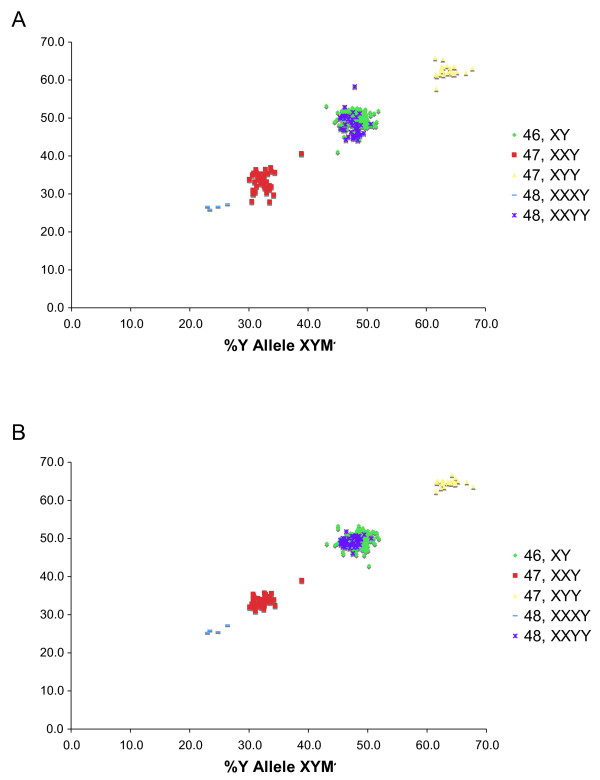
**Percent Y allele signal of XYM2 (A) and XYM3 (B) plotted against the percent Y allele signal of XYM1 for 323 DNA samples from male subjects grouped by karyotype.** For each marker, the percent Y allele signal is the ratio of signal from the Y-chromosome specific allele divided by the total signal from both the Y- and X-chromosome specific alleles, expressed as a percentage.

Data generated with the three XYM markers were analyzed for the 323 DNA samples from males with karyotype-confirmed sex chromosome aneuploidies (n = 117) or 46,XY (n = 206). Scatter plots of the percent Y allele signal for both the XYM2 and XYM3 markers versus the percent Y allele signal of the XYM1 marker are shown in Figure 3. For all three XYM markers, the data for individuals with the 48,XXXY, 47,XXY, and 47,XYY karyotypes were tightly clustered and distinguishable from males with a 46,XY karyotype. As expected from the known Y:X chromosome ratio, the percent Y allele signals for the subjects with 48,XXYY karyotype overlapped with those for the 46,XY males. The percent Y allele signal for both XYM2 and XYM3 was strongly correlated with XYM1, with correlation coefficients (r^2^) of 0.90 and 0.94, respectively. Table 1 summarizes the statistical data calculated for the measured percent Y allele signal of each marker for samples grouped according to karyotype; the average percent Y allele signal for each group was close to the predicted value based on the known karyotype.

### Detection of 47,XXY (KS), 48,XXXY and 47,XYY syndromes

**Figure 4:**
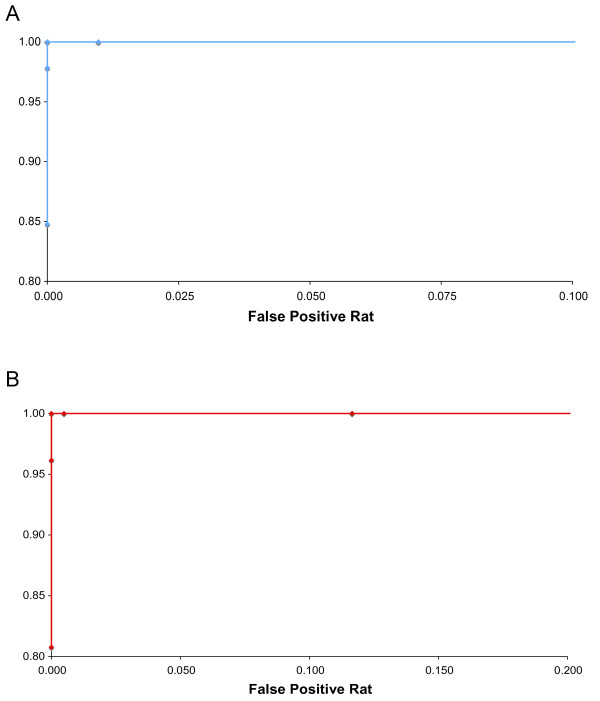
**Receiver operator characteristic curves for detection of 47,XXY (KS) and 48,XXXY (A) and 47,XYY syndromes (B).** The average of the three XYM marker data points for each sample was calculated and compared to the detection thresholds. The threshold for detecting XXY and XXXY syndromes was varied by 1% increments from 34-48% Y allele signal; that for detecting XYY syndrome was incremented by 1% from 49-63%. The sensitivity (TP/(TP + FN)) and false positive rate (FP/(FP + TN)) were calculated for each value of the appropriate threshold. For XXY and XXXY, true positives (TP) have either a 47,XXY or 48,XXXY karyotype and average Y allele signal less than the threshold; false negatives (FN) have the same karyotypes and average Y allele signal greater than or equal to the threshold. True negatives (TN) for XXY and XXXY are samples with a 46,XY karyotype and average Y allele signal greater than or equal to the threshold; false positives (FP) have a 46,XY karyotype and average Y allele signal below the threshold. For XYY syndrome, true positives have 47,XYY karyotype and average Y allele signal greater than the threshold; false negatives have the same karyotype and average Y allele signal less than or equal to the threshold. True negatives for XYY are samples with a 46,XY karyotype and average Y allele signal less than or equal to the threshold; false positives have a 46,XY karyotype and average Y allele signal above the threshold.

To identify 47,XXY, 48,XXXY, or 47,XYY karyotypes, receiver operator characteristic (ROC) curves were constructed by varying the percent Y allele signal lower and upper thresholds for samples scored as normal 46,XY karyotype (Figure 4). For this analysis, the average percent Y allele signal for the three XYM markers was calculated; the lower threshold was increased from 34% to 48% by 1% increments while sensitivity and false positive rates for detecting 47,XXY (KS) and 48,XXXY karyotypes were calculated. All samples with either 47,XXY or 48,XXXY karyotypes and average percent Y allele signals less than the lower threshold value were classified as true positives. False negative samples had the same karyotypes and the average percent Y allele signal above the threshold. True negatives were defined as a 46,XY subject with the average percent Y allele signals greater than the lower limit, while a false positive was defined as having the average percent Y allele signal less than the lower limit.

Conversely for 47,XYY syndrome, the upper threshold was examined from 49% to 63% by 1% increments, and sensitivity and false positive rates were calculated. True positives had 47,XYY karyotype and an average percent Y allele signal above the upper threshold; any 47,XYY sample with the average percent Y allele signal below the upper limit was scored as false negative. True negatives (46,XY) had the average percent Y allele signal less than or equal to the upper limit; false positives had the average percent Y allele signal above the upper limit.

Examination of the ROC curves indicated that a percent Y allele signal scoring threshold of 43% for 47,XXY and 48,XXXY syndromes and 57% for 47,XYY syndrome yielded 100% sensitivity with a 0% false positive rate. Combining the two thresholds gave a percent Y allele signal range of 43-57% for 46,XY samples. Separate analysis of the individual XYM marker data and the median of the three XYM values generated ROC curves which overlapped the curve generated with the average XYM values; thresholds of 43% and 57% allow the detection of KS and 47,XYY syndrome with 100% sensitivity and specificity using the data from either each individual marker or the median value.

### Detection of 48,XXYY syndrome

**Figure 5:**
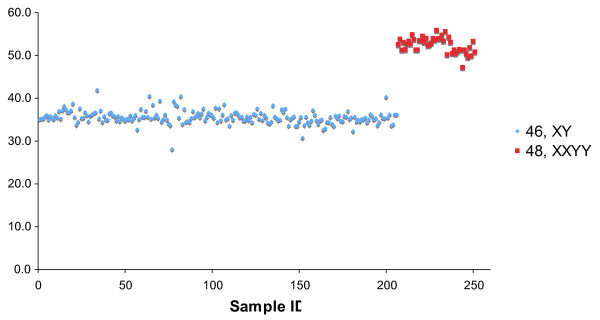
**Average percent X allele signal of four XA markers versus sample ID for male samples with 46,XY and 48,XXYY karyotypes.** The average percent X allele signal is the average for all four markers of the ratio of signal from the X-chromosome specific allele divided by the total signal from both the X-chromosome and autosome specific alleles, expressed as a percentage.

**Figure 6:**
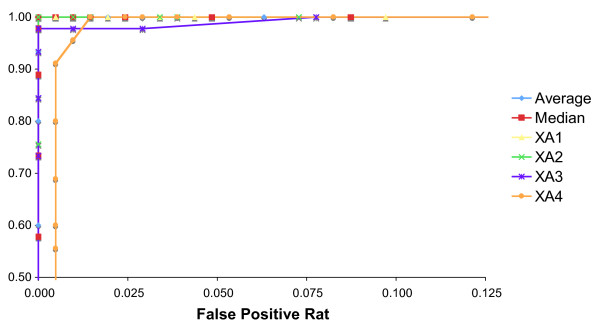
**Receiver operator characteristic curves for detection of 48,XXYY syndrome.** The average and median of the four XA marker data points for each sample was calculated: either the average, median or individual marker data was compared to the threshold for detecting 48,XXYY syndrome. The threshold was varied by 1% increments from 33-52% X allele signal. The sensitivity (TP/(TP + FN)) and false positive rate (FP/(FP + TN)) were calculated for each value of the appropriate threshold. True positives (TP) have a 48,XXYY karyotype and X allele signal greater than the threshold; false negatives (FN) have the same karyotype and X allele signal less than or equal to the threshold. True negatives (TN) have a 46,XY karyotype and X allele signal less than or equal to the threshold; false positives (FP) have a 46,XY karyotype and X allele signal above the threshold.

As noted above, the percent Y allele signals from 48,XXYY individuals (n = 45) and 46,XY males (n = 206) showed nearly complete overlap (Figure 3 and Table 1). To distinguish these karyotypes, four XA markers were amplified by PCR and the resulting products examined by Pyrosequencing. Figure 5 is a plot of the average percent X allele for the four XA markers graphed versus the sample ID. The average percent X allele signal differed for the samples with 48,XXYY and 46,XY karyotypes and was close to the expected values of 50% and 33.3%, respectively, based on the known chromosome ratios (Table 2). ROC analysis (Figure 6) performed by comparing the average percent X allele for the four XA markers with a threshold varying from 33-52% in 1% increments indicated that a threshold of 43% gave 100% detection of 48,XXYY karyotype with a 0% false positive rate.

As a confirmatory approach to distinguish males with 48,XXYY and 46,XY karyotypes, 18 X-linked markers were PCR amplified and the amplicons subjected to Pyrosequencing using DNA from all 48,XXYY males. The relative allele strength for each of the X-chromosome specific markers was scored as homozygous, heterozygous, or out-of-range as described for a Turner Syndrome assay [16]. Of the 45 individuals with the 48,XXYY karyotype, 43 demonstrated definitive evidence for the presence of two distinct X-chromosomes, with the number of heterozygous markers ranging from 4 to 12 out of 18 total. Thus, most 48,XXYY individuals either inherit one X-chromosome from each parent or inherit two distinct X chromosomes as a result of nondisjunction in maternal meiosis I. The remaining two individuals were homozygous for all 18 markers, and therefore, appear to have inherited both X chromosomes from their mother due to nondisjuction in maternal meiosis II or as a result of nondisjunction of a 46,XY embryo in early mitotic cell divisions. Thus, for the latter group, only signal from the XA markers distinguished them from 46,XY individuals.

### Analysis of Coriell samples with a 47,XXY karyotype

In an additional test of the sex chromosome aneuploidy assay, three XYM markers were measured on DNA obtained from immortalized lymphocyte cultures of 16 individuals with a 47,XXY karyotype (Coriell Institute of Medical Research). For 15 of 16 individuals, the percent Y allele signal for all three markers clustered tightly around the expected 33.3%. One individual had percent Y allele signals between 53.6 and 59% and is known to have a complex mosaic karyotype: 47,XXY [17].ish X (DXZ1x2).ish Y (SRYx1)/47,XYY [28].ish X (DXZ1x1).ish Y (SRYx2)/46,XY [5].ish X (DXZ1x1).ish Y (SRYx1), with prominent contributions from both 47,XXY and 47,XYY cell populations. Based on the mosaic estimate, this subject is expected to have percent Y allele signals near 50%.

### Analysis of buccal swab samples

As a final test for assay performance, buccal swabs were obtained from 29 males with known karyotypes. Buccal cell DNA was extracted and all seven markers amplified by PCR. Following Pyrosequencing, the data from all 29 samples were classified using the previously described threshold values for the XYM and XA markers. Table 3 presents the aggregate statistical data for the average allele signal of the buccal swab samples grouped by karyotype. Buccal swab samples demonstrated 100% sensitivity and specificity for detection of male sex chromosome aneuploidies.

**Table 3:** Statistical values calculated from the average percent allele signals of the XYM and XA marker sets for buccal swab samples grouped together by karyotype

**Karytoype**	**47, XXY (n = 8)**		**47, XYY (n = 3)**		**46, XY (n = 5)**		**48, XXYY (n = 13)**	
**Marker Set**	**Average Y Allele XYM**	**Average X Allele XA**	**Average Y Allele XYM**	**Average X Allele XA**	**Average Y Allele XYM**	**Average X Allele XA**	**Average Y Allele XYM**	**Average X Allele XA**
Average	35.1	52.1	65.2	36.3	50.6	38.1	50.1	52.3
Median	35.0	51.5	65.1	36.1	50.7	37.7	50.3	52.1
Std Dev	0.9	1.7	0.2	0.9	0.8	1.3	1.0	1.9
Maximum	36.8	54.3	65.4	37.3	51.6	39.5	52.1	56.3
Minimum	34.0	50.2	65.0	35.6	49.6	36.6	48.0	49.7

## Discussion

We report the development of a rapid, high-throughput Pyrosequencing assay for detecting sex chromosome aneuploidies in males. The assay initially interrogates three XYM markers, yielding the percent Y allele signal which is directly related to the Y:X chromosome ratio. Next, to distinguish 46,XY and 48,XXYY karyotypes, the assay utilizes four XA markers to determine the X:A ratio. Using this approach, our assay identifies males with 47,XXY, 47,XYY, 48,XXXY and 48,XXYY karyotypes at 100% sensitivity and specificity (Table 4).

**Table 4:** Sensitivity and false positive rate for detection of sex chromosome aneuploidies in males

**Karyotype**	**Total Samples**	**Sensitivity**	**False Positive Rate**
47,XXY (KS)	65	100%	0%
47,XYY	29	100%	0%
48,XXXY	4	100%	0%
48,XXYY	58	100%	0%

Combination of results for DNAs isolated from buccal swabs (n = 29) or obtained from Colorado Children’s Hospital, UC Davis MIND Institute, Children’s Hospital Boston, Yale University and Coriell Institute (n = 338). One Coriell sample was omitted from this table due to its complicated mosaic karyotype: 47,XXY [17].ish X (DXZ1x2).ish Y (SRYx1)/47,XYY [28].ish X (DXZ1x1).ish Y (SRYx2)/46,XY [5].ish X (DXZ1x1).ish Y (SRYx1).

Previous studies of the parental origin of the sex chromosomes in males with 48,XXYY are limited to a total of eight individuals [17-21]. These studies concluded that the extra sex chromosomes are paternally derived, resulting from two sequential nondisjunction events in meiosis I and II of spermatogenesis. Our data for 96% of subjects with 48,XXYY (n = 43) are consistent with this mechanism since the DNA samples demonstrated heterozygosity for between 4 and 12 of a total 18 X-linked biallelic SNP markers. However, the data cannot rule out an alternative mechanism whereby the additional X chromosome is maternally derived from nondisjunction in meiosis I of oogenesis and the supernumerary Y is due to nondisjunction in meiosis II of spermatogenesis. Only detailed molecular genetic analysis of the parents of 48,XXYY males can ascertain the relative contribution of these two mechanisms; still, the statistical likelihood of an X aneuploid oocyte being fertilized by a Y aneuploid sperm is quite low. The remaining 4% of subjects with 48,XXYY (n = 2) were shown to have identical X chromosomes due to complete homozygosity of the 18 X-linked markers and thus likely result from nondisjunction during early mitotic divisions of a 46,XY embryo. Alternatively, but less likely, this subset of individuals may result from nondisjunction in meiosis II of both maternal and paternal gametes. The current study has a large enough population (n = 45) to detect this novel mechanism for the chromosomal origin of the supernumerary sex chromosomes in 48,XXYY males.

Our Pyrosequencing based assay is robust and readily interpretable allowing the reliable detection of male sex chromosome aneuploidies with 100% sensitivity and specificity. This particular methodology serves as a rapid, high-throughput screen, and the accuracy of detection for KS and other sex chromosome aneuploidies translates to an extremely low likelihood of discrepant karyotypic analysis if utilized for confirmation. The assay may be completed in 8–10 hrs. which is considerably faster than the time required for either fluorescent in situ hybridization (FISH) or karyotype analysis. Individuals with KS may present clinically at many points during their lifetime [1], and yet, because of variable and often subtle phenotype, they are not recognized and in most cases, never diagnosed [22]. The current assay provides a non-invasive molecular test applicable for rapid diagnosis, thus allowing for earlier assessments and interventions in all facets of therapy for KS, 47,XYY, 48,XXYY and 48,XXXY, including androgen replacement and cognitive and behavioral treatments [23].

For male children suspected of KS or another sex chromosome aneuploidy, the ability to make the diagnosis using DNA isolated from buccal swabs is an advantage over invasive, often traumatic, blood testing. Diagnosis during infancy/childhood, especially for KS, allows for early interventional speech/language therapy and educational planning, as well as promotion of physical activity to inhibit the development of dyspraxia. Endocrine monitoring and early management can be instituted to eventually support puberty, preserve fertility, and determine the timing of androgen replacement [23]. With respect to 47,XYY syndrome, rapid and efficient detection similarly permits initiation of appropriate cognitive and behavioral therapy. Current trials of pharmaceuticals for general developmental disorders in male children that overlap with KS and other supernumerary X chromosome syndromes may also benefit from diagnostic specificity for these relevant aneuploidies.

Even making the delayed diagnosis of KS or other sex chromosome aneuploidies in adulthood offers specific treatment goals for their related complications. KS is one of the most frequent causes of male infertility [24]. For adult males being evaluated for failure to conceive, making this diagnosis earlier offers specific and better options to preserve fertility [25], and this rapid methodology may decrease the stress and anxiety associated with waiting for the diagnosis of KS (or other sex chromosome aneuploidy) by karyotyping. As with male children, making the diagnosis of KS in adulthood is also important for instituting specific endocrine therapies to prevent gynecomastia and osteopenia [23].

The clinical application of this sensitive and specific Pyrosequencing based assay will enable the rapid, efficient and high-throughput detection of sex chromosome aneuploidies in males and allow for early, appropriate assessment and therapeutic interventions for individuals with these common but under-diagnosed genetic conditions.

## Competing interests

Karl Hager, Kori Jennings & Seiyu Hosono are employees of JS Genetics, Inc.. Karl Hager, Seiyu Hosono, Jeffrey R. Gruen, Scott A. Rivkees, and Henry M. Rinder hold equity interest in JS Genetics, Inc.. Susan Howell and Nicole R. Tartaglia have no competing interests.

## Authors’ contributions

KH contributed to study design, developed the hypothesis, analyzed data, and wrote the manuscript; KJ analyzed data and contributed to writing of the manuscript; SH contributed to study design and writing of the manuscript; SH contributed to study design, writing of the manuscript, and collection of patient samples; JG contributed to study design and writing of the manuscript; SR contributed to study design and writing of the manuscript; NT contributed to study design, writing of the manuscript, and collection of patient samples; HR developed the hypothesis, analyzed data, and contributed to study design and writing the manuscript. All authors read and approved the final manuscript.
